# European Correspondence

**Published:** 1853-12

**Authors:** 


THE NORTH-WESTERN

MEDICAL AND SURGICAL JOURNAL.

NEW SERIES.

VOL. II. DECEMBER, 1853. NO. 8.

### ORIGINAL COMMUNICATIONS.

ART. 1.—*European Correspondence.*

LONDON, *October 27th*, 1853.

DR. JOHNSON: Dear Sir: According to promise, I sit down to give you a sketch of men and things in Europe, medical and surgical, as I might see them. Having but a day in London in passing, I improved it by a visit to the Museum of the Royal College of Surgeons, founded by John Hunter. I was informed by the porter that the Museum was closed, and could not be seen for several days—the closing being for the purpose of transferring the specimens to the new Hall, and re-arranging them in the old. On sending my card, with a note of introduction from a member, I was, as a foreigner, readily admitted, and Mr. Quickett, the Curator, and Mr. Luke, the President, immediately met me in the Hall, and in the most obliging manner pointed out those objects of interest for which I particularly inquired. Unfortunately the disarrangement of specimens did not allow him to point out to me more than two illustrating the effects of foreign bodies placed in contact with bone, the special object of my search, and not one of a bone, the union of which after fracture had been effected by means of the seton, wires, or other foreign bodies. Disappointed in this respect, I was not so in others, the Museum being rich in all the more ordinary specimens. Hunter's experiments on the production of necrosis by separating the periosteum, is shown by numerous preparations of bones of the Ass, in as perfect a state of preservation as the day they were deposited, side by side with pieces of wood, from parts of which the bark had been removed, where the wood, like the bone, was dead. The analogy of the periosteum and the bark was a favorite idea of Hunter.

Hunter's experiments on regeneration of tendon are perfectly shown by his preparations.

Casually pieces of the intestines of Napoleon were pointed out, but the story that they had been demanded by the French Government, as part of the remains, was discredited.

The knife swallowed by a conjurer, and which remained in the stomach for several years, until half rusted away, without much inconvenience, when it produced death by a thrust in the side from an upset of the cart, was also seen.

The duodenum of the woman who died from swallowing bent pins, is preserved perfectly filled with them, as well as several ounces taken from the stomach and intestines, and preserved in bottles.

The collections of Sir Astley Cooper and Mr. Liston have, within a few years been added to this Museum; among the former a case of fracture of the neck of the femur, entirely within the capsule and entirely united by bone, may be noticed. This, it is said, was kept in a private collection, and never shown or alluded to by Sir Astley, although it was known to be in his possession having been presented by a friend.

Among the improvements recently introduced in the College, is a lecture room, arranged for the purpose of teaching microscopic anatomy, the first of the kind ever made use of. It consists of two benches of semi-circular form, with tables in front, upon which the microscope runs, as upon a railway. These are connected with the table of the lecturer, so that the microscope can pass from and to it. The advantages of this arrangement are, that one microscope suffices for a class, and that thirty or forty objects can thus be exhibited to a class in a single hour. This has been introduced by Mr. Quickett, the Professor of Microscopic Anatomy, who has also added to the Museum about 8,000 specimens to be viewed by the microscope. These are preserved between strips of glass with turpentine in the usual manner. The specimens preserved in fluids are placed in spirit, about five degrees above proof; the bottles are closed by placing over them first, a piece of bladder, varnished, over that a piece of thin lead, lined with tin foil, then another piece of bladder, varnished. The cholesterine which dissolves renders the liquid slightly turbid at a low temperature, but when slightly warmed, it is perfectly transparent and unchanged by time.

The new Hall, into which the specimens are being removed in part, is of vast dimensions and beautiful proportions. It was erected principally by funds of the College, the Government appropriating but a small sum for that purpose. The library contains 30,000 volumes, conveniently arranged in a very fine Hall. It does not contain a single manuscript of Hunter, of all the numerous ones which he left at his death, but the history of their destruction or publication by a high officer of the College, as his own, has never, as yet, been more than partly given to the public. Those best informed seem to know but little of the family and personal history of Hunter. He is buried, it is said, in St. Martin's Church. He left, at his death, a son and a daughter, but whether his descendants are living, I was not able to ascertain. Such men as James Watt and John Hunter are not ennobled in England.

The governor's room contains a portrait of Hunter, by Sir Joshua Reynolds, busts of Hunter, Cline, Abernethy, Sir Astley Cooper, etc. The bust of Hunter shows his head to have been massive, very broad from ear to ear and antero-posteriorly, but proportionally, not high. Cline's is not unlike it in this respect.

The amphitheatre of the College is very handsome and spacious, capable of seating about 500 persons.

Thanks to the temporary closure of the Museum, which enabled me, instead of wandering through the Halls alone, to see the collection under the guidance of its distinguished President and the

Curator, to pass an hour delightfully in interchange of views with them on professional subjects. I hope, on my return from Paris in the Spring, to spend a month in London, and make myself as much acquainted as possible with its different medical institutions and hospitals, when I may note for your readers such progressive tendencies as I may observe. B.

